# The Systematic Review Toolbox: keeping up to date with tools to support evidence synthesis

**DOI:** 10.1186/s13643-022-02122-z

**Published:** 2022-12-01

**Authors:** Eugenie Evelynne Johnson, Hannah O’Keefe, Anthea Sutton, Christopher Marshall

**Affiliations:** 1 Population Health Sciences Institute, Newcastle University, Newcastle upon Tyne, UK; 2 NIHR Innovation Observatory, Newcastle University, Newcastle upon Tyne, UK; 3 School of Health and Related Research (ScHARR), The University of Sheffield, Sheffield, UK; 4 York Health Economics Consortium, University of York, York, UK

**Keywords:** Systematic review, Evidence synthesis, Support, Tools, Guidance, Software

## Abstract

**Background:**

The Systematic Review (SR) Toolbox was developed in 2014 to collate tools that can be used to support the systematic review process. Since its inception, the breadth of evidence synthesis methodologies has expanded greatly. This work describes the process of updating the SR Toolbox in 2022 to reflect these changes in evidence synthesis methodology. We also briefly analysed included tools and guidance to identify any potential gaps in what is currently available to researchers.

**Methods:**

We manually extracted all guidance and software tools contained within the SR Toolbox in February 2022. A single reviewer, with a second checking a proportion, extracted and analysed information from records contained within the SR Toolbox using Microsoft Excel. Using this spreadsheet and Microsoft Access, the SR Toolbox was updated to reflect expansion of evidence synthesis methodologies and brief analysis conducted.

**Results:**

The updated version of the SR Toolbox was launched on 13 May 2022, with 235 software tools and 112 guidance documents included. Regarding review families, most software tools (*N* = 223) and guidance documents (*N* = 78) were applicable to systematic reviews. However, there were fewer tools and guidance documents applicable to reviews of reviews (*N* = 66 and *N* = 22, respectively), while qualitative reviews were less served by guidance documents (*N* = 19). In terms of review production stages, most guidance documents surrounded quality assessment (*N* = 70), while software tools related to searching and synthesis (*N* = 84 and *N* = 82, respectively). There appears to be a paucity of tools and guidance relating to stakeholder engagement (*N* = 2 and *N* = 3, respectively).

**Conclusions:**

The SR Toolbox provides a platform for those undertaking evidence syntheses to locate guidance and software tools to support different aspects of the review process across multiple review types. However, this work has also identified potential gaps in guidance and software that could inform future research.

**Supplementary Information:**

The online version contains supplementary material available at 10.1186/s13643-022-02122-z.

## Introduction

The Systematic Review Toolbox (SR Toolbox) was developed in 2014 by Christopher Marshall (CM) as part of his PhD surrounding tools that can be used to support the systematic review process within software engineering [1]. Whilst originally developed for the field of computer science, the methodologies for conducting systematic reviews and evidence synthesis are applicable across disciplines. Therefore, the scope of the SR Toolbox was expanded to include health topics. Its aim is to assist researchers by providing an open, free and searchable web-based catalogue of tools and guidance papers that assist with various tasks within the systematic review and wider evidence synthesis process. The SR Toolbox is regularly maintained by conducting a specialised search on MEDLINE, before being screened according to a defined inclusion and exclusion criteria by a single Editor, checked by a second editor (see Additional file 1: Supplementary Material). Guidance and software tools that meet the eligibility criteria are added to the SR Toolbox on a rolling basis.

In January 2022, the SR Toolbox website gained approximately 28,500 hits and 6100 visits from around 4500 unique visitors, showing the popularity of the platform and its potential reach to researchers looking to find tools and guidance for use within evidence syntheses. However, since the initial launch of the SR Toolbox in 2014, there has been an increase in the number and types of evidence syntheses being produced. Many systematic review typologies and taxonomies had been developed since the SR Toolbox’s inception, including large numbers of review types. For example, Booth et al. (2016) identified 22 review types [2], Cook et al. (2017) identify 9 [3], while the typology by Munn et al. (2018) suggested there were 10 different review types [4].

More recently, a taxonomy proposed by Sutton et al (2019) incorporating research from several other previously published works suggests that 48 review types exist [5], which can be broadly categorised into seven review “families”:

- Traditional reviews (that tend to use a purposive sampling approach as opposed to a systematic approach);
- Systematic reviews;
- Review of reviews;
- Rapid reviews;
- Qualitative systematic reviews;
- Mixed-methods reviews; and
- Purpose-specific reviews (i.e. reviews that are tailored to individual needs, such as Health Technology Assessment).

In the version of the SR Toolbox prior to 2022, the ability to search by review type was limited and not reflective of the expanding evidence synthesis landscape. The SR Toolbox’s ability to suggest support for the varying demands of different review types was therefore limited.

Additionally, although there is now a large array of tools available to support the process of conducting systematic reviews and other forms of evidence syntheses, a potential barrier to adoption includes inexperience of some of the underlying principles of tools, such as machine learning [6]. In the iteration of the SR Toolbox maintained until 2022, software tools were searchable according to their underlying approach (e.g. text mining, machine learning, visualisation), discipline (healthcare, social sciences, software engineering or multidisciplinary), and their financial cost (e.g. completely free or payment required). “Other” tools were only searchable by discipline and type (e.g. guideline, reporting standards). As such, for those with less experience or knowledge of the processes underpinning software tools, effective searching of the SR Toolbox could potentially be challenging.

We therefore set out to update the SR Toolbox interface, so it continues to be able to respond to the needs of users within a changing and continually developing evidence synthesis landscape, as well as being more accessible to a wide variety of researchers. In this paper, we describe our methods for reconstructing the platform by conducting a mapping exercise of all tools within the SR Toolbox to re-categorise them and check their validity. In addition, we also describe a brief analysis based on the mapping exercise to identify review types and processes that are both well-served and underserved by the tools currently contained within the platform.

## Methods

### SR Toolbox update methods

In February 2022, we embarked on a mapping exercise of all software and other tools indexed within the SR Toolbox to inform the restructuring of the platform. A coding tool was developed in Excel to extract data relevant to each tool indexed within the SR Toolbox to that point. Domains were either completed using free text or ticked using a check box. Details of domains assessed and how they were coded are detailed in Table 1.

**Table 1:** Coding framework for mapping exercise

Domain	Contents	Type of data
Tool name	• Tool name	Free text
Tool characteristics	• Tool summary • Link to tool or paper • Additional publication links • Last known tool update	Free text
Tool type	• Guidance (e.g. papers outlining specific methodologies for evidence syntheses or individual stages within evidence syntheses) • Software	Check box
Review family/type	• Systematic • Rapid • Qualitative • Scoping • Mapping • Mixed methods • Review of reviews • Other (e.g. diagnostic test accuracy, prognostic)	Check box
Review stage	• Protocol • Search • Screening • Data extraction • Quality assessment • Synthesis • Reporting • Reference management • Stakeholder engagement	Check box
Cost	• Free • Free trial • Free version available • Payment required • Open access (for papers) • Not open access (for papers)	Check box
Date added to the SR Toolbox	• Date added to the SR Toolbox	Free text

Part of the coding framework was adapted from the review family taxonomy proposed by Sutton et al. (2019) [5]. However, we did not include traditional reviews and purpose-specific reviews within the mapping exercise. This is because traditional reviews as described by the Sutton taxonomy were not considered systematic enough to be within scope for the SR Toolbox, while purpose-specific reviews were too broad and (potentially) too diverse to include in a systematic manner, as they include a wide variety of evidence syntheses including scoping reviews, mapping reviews and Health Technology Assessment [5]. Although both scoping reviews and mapping reviews are part of the purpose-specific family within the Sutton taxonomy [5], we separated these into their own categories. This is because it has been noted that scoping reviews are growing in number [7], while mapping reviews are becoming increasingly conducted as a way of visually representing the breadth of a body of evidence, despite being rare until as recently as 2010 [8]. Mapping reviews can also be considered distinct from scoping reviews, as although both present a broad overview of evidence relating to a topic, they are highly visual in nature [9]. Furthermore, it has been posited that scoping reviews can act as a precursor to a predefined systematic review, whereas mapping reviews may aim to identify research areas for systematic review or gaps in the evidence base [5].

All records currently contained within the SR Toolbox up to February 2022 (*n* = 352) were manually extracted and coded according to the framework by a single reviewer (EEJ). The same reviewer checked all current records to ensure that hyperlinks were not broken and that tools still appeared to be active. If links to software tools were no longer active and could not be located elsewhere, these were excluded from the mapping exercise and, subsequently, the SR Toolbox (*N* = 5). Tools and guidance could be coded to more than one review family and more than one stage of a review, where appropriate. A second reviewer (HOK) checked a small percentage of the records coded for accuracy before the spreadsheet was imported into a Microsoft Access database.

**Fig. 1:**
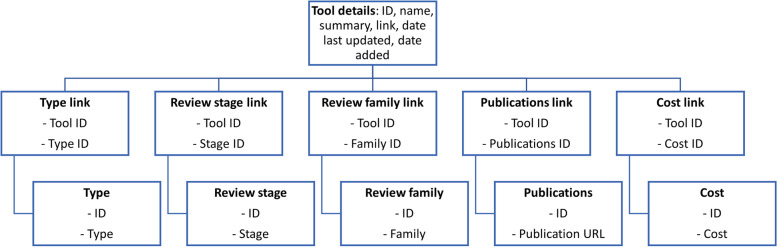
Diagram of Microsoft Access framework

Microsoft Access databases are relational, meaning that relationships can be built between tables. We included a table for tool details, tool type, review stage, review family, publications, and cost. The tool details table acted as the main reference point, with all other tables being related to it via interim linker tables (Fig. 1).

The tables contained in the local database in Access were exported as separate CSV files, then imported using phpMyAdmin to create the same database, online, in MySQL. Custom structured query language (SQL) statements, which accounted for any combination of user query, were hard coded into the website’s hypertext preprocessor (PHP) scripts. Furthermore, the graphical user interface that facilitates users in running advanced searches was updated to reflect the updated database and new tool categories.

### Analysis methods

We undertook a basic analysis of the different software tools and guidance documents included within the SR Toolbox up to February 2022 in order to assess: what review families were being covered by the included tools; what review stages and aspects were being covered by the included tools; how up to date included software tools are; and the trajectory of research for guidance and reporting documents relating to evidence syntheses.

Using the same coding document developed in Excel for the mapping exercise described above, we filtered the spreadsheet to contain either relevant software tools or relevant guidance so they could be analysed as separate entities. From here, we tabulated the number of times tools or guidance documents were checked against each review family or review stage. We also added an additional column to the spreadsheet to indicate where tools or guidance documents could be applicable to multiple review families or multiple review stages; these were manually coded within the spreadsheet. The numbers tabulated from each of these exercises were used to create tables and graphs demonstrating the volume of tools in each category.

## Results

### SR Toolbox update

At the time of updating the SR Toolbox interface, there were 235 software tools and 112 guidance or reporting documents included within the platform. The new SR Toolbox interface was launched on 13 May 2022.

### Analysis results

Table 2 documents the relevance of guidance documents and software tools contained within the SR Toolbox to different review families. Of the 235 software tools and 112 guidance documents currently contained within the SR Toolbox, 215 software tools (91.5%) and 61 guidance documents (54.5%) can be applicable to multiple review families. Most software tools (*N* = 223) and guidance documents (*N* = 78) are applicable to systematic reviews, though far less are applicable to reviews of reviews (*N* = 66, 28.1% and *N* = 22, 19.6% respectively). Qualitative reviews were slightly better served in terms of software tools (*N* = 108, 46%), but were the most under-served review family in terms of guidance documents (*N* = 19, 17%).

**Table 2:** Relevance of software tools and guidance documents included in the SR Toolbox to different review families

Review family	Software tools (***N*** = 235)	Guidance (***N*** = 112)
Systematic reviews	223	78
Rapid reviews	190	48
Qualitative reviews	108	19
Scoping reviews	142	44
Mapping reviews	144	45
Mixed methods	168	27
Reviews of reviews	66	22
Other review (e.g. DTA)	188	47
Multiple review types	215	61

Key: *DTA* diagnostic test accuracy, *SR* systematic review

Table 3 shows the amount of software tools and guidance contained within the SR Toolbox at the time of update in relation to what stage of the review production process they assist with. Seventy-five (32%) of the software tools were applicable to more than one review production stage, while only 16 (14.3%) guidance documents were applicable to multiple stages of the process. Guidance documents within the SR Toolbox are currently dominated by research relating to quality assessment (*N* = 70; 62.5%), followed by guidelines for reporting reviews (*N* = 26; 23.2%). There appears to be a paucity of software tools (*N* = 2; 0.9%) and guidance (*N* = 3; 2.7%) that relates to stakeholder engagement within the review process.

**Table 3:** Relevance of software tools and guidance documents included in the SR Toolbox to different review production stages

Review stage	Software tools (***N*** = 235)	Guidance (***N*** = 112)
Protocol development	24	12
Searching	84	12
Study selection/screening	45	4
Data extraction	57	6
Quality assessment	34	70
Synthesis	82	7
Reporting	37	26
Reference management	37	N/A
Stakeholder engagement	2	3
Multiple review stages	75	16

Key: *SR* systematic review

**Fig. 2:**
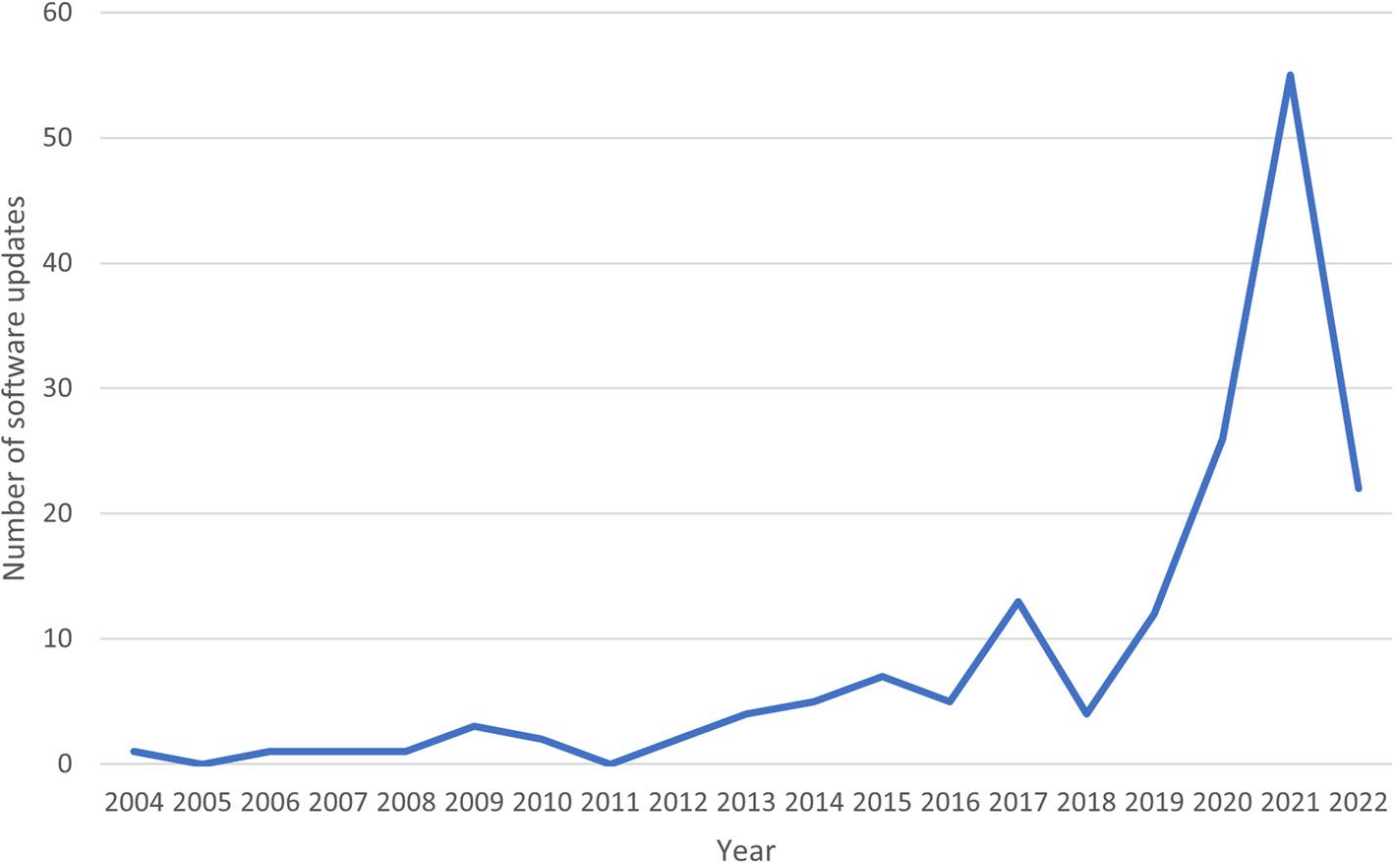
Number of updates for software tools included in the Toolbox by year (*N* = 164)

Figure 2 shows how up to date the software tools included within the SR Toolbox are. Most of the tools for which a new version was available have been updated within the past 4 years up to and including the first quarter of 2022 (*N* = 115), with the most updates occurring in 2021 (*N* = 51). However, although this is suggestive that most of the tools included in the SR Toolbox could be considered up to date, there were 71 software tools where we could not identify the latest update date (30.2% of all included software tools). We therefore cannot be certain that a relatively large proportion of software tools within the SR Toolbox are up to date.

**Fig. 3:**
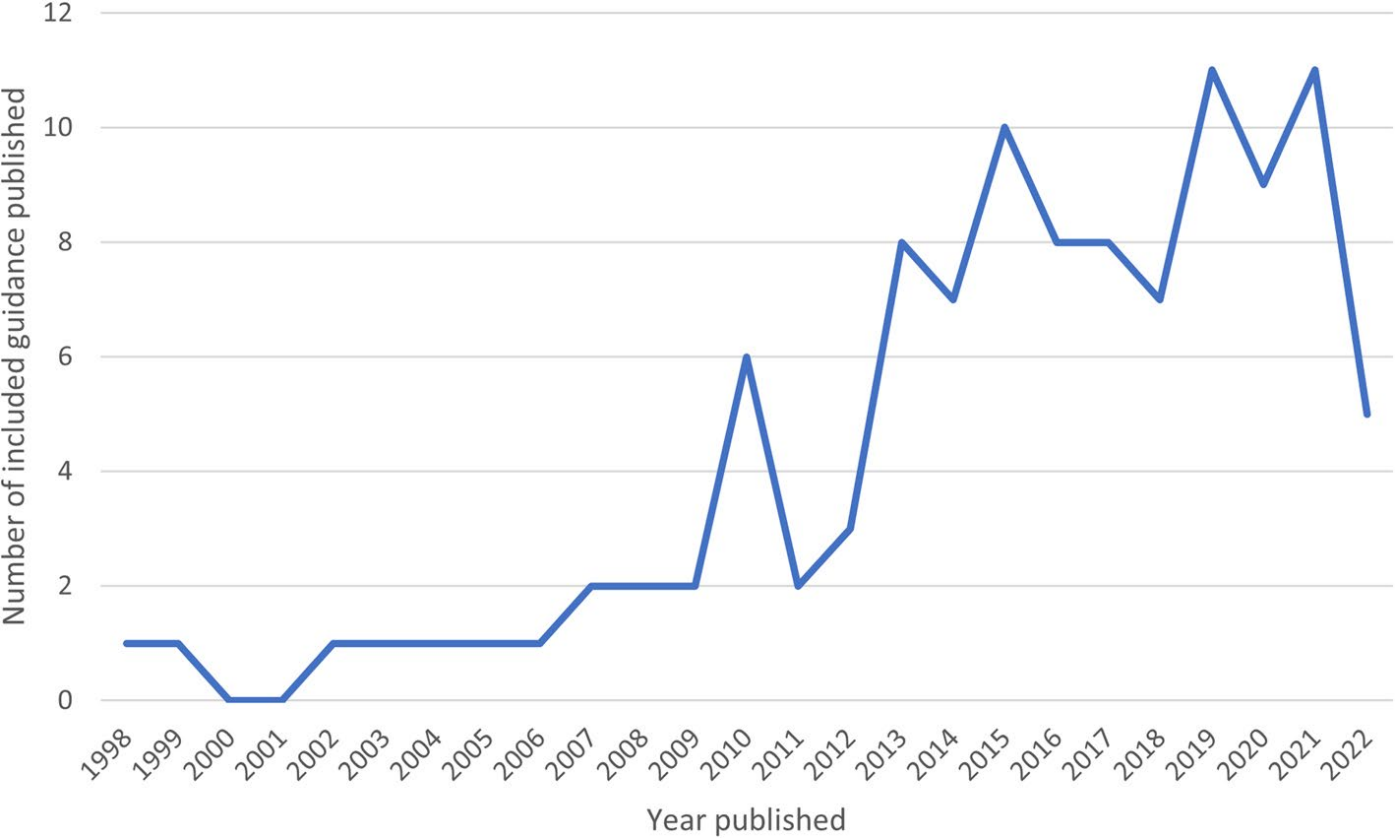
Number of guidelines and reporting frameworks included in the Toolbox published by year (*N* = 108)

Similarly, Fig. 3 shows the amount of guidance and reporting tools included within the SR Toolbox by the year in which they were published. Although it was not clear when four of the guidance documents were originally published or updated, this only represents a small proportion of the guidance included within the Toolbox (3.6%). The earliest guidance publication date included within the SR Toolbox dates to 1998. However, of all the guidance and reporting documents included within the SR Toolbox, the majority have been published since 2015 (63.9%). The greatest number of guidance documents or reporting tools were published in 2019 and 2021 (11 per year). Before beginning the SR Toolbox updating exercise, we had already identified five new eligible guidance and reporting documents published in 2022. These data suggest that there has been a steady increase in the number of publications offering guidance and reporting standards relating to systematic reviews and wider evidence syntheses since 1998 and the trajectory of publications in this area has been particularly high since 2015.

Most of the included software tools are free to use (181/235, 77%). Of the 21 software tools that required payment, 12 had a free trial available and 3 had a free version available. Similarly, most of the guidance documents are open access (96/112, 85.7%).

## Discussion

### Summary of main results

The update of the SR Toolbox aims to provide a simple and easily navigable interface for researchers to discover guidance and software tools to help conduct systematic reviews and wider evidence syntheses projects. The new structure of the SR Toolbox, which incorporates the ability to search by review family and review stage, has been developed and implemented to make it easier for researchers and other stakeholders with less familiarity and experience with the underlying computational concepts of tools. Stakeholders should be more able to identify and access software and guidance that may assist them with their evidence syntheses projects.

Our brief analysis of tools included in the platform up to February 2022 suggests that many software tools and guidance documents currently within the SR Toolbox can potentially be applicable to multiple review families, though reviews of reviews and qualitative reviews may currently be less well served. Guidance documents largely focus on methods for critical appraisal, followed by reporting guidelines, with far fewer publications surrounding other aspects of the review production process. Additionally, software tools to support the systematic review process may be mostly well-maintained and up to date, though there is some uncertainty surrounding this. The trajectory of guidance and reporting frameworks for evidence syntheses being published has been steadily increasing and has seen a particular increase since 2015.

### Strengths and limitations of this work

Well-defined categories were used to map the guidance and software tools, based on widely accepted published standards [5]. These categories were agreed upon by highly experienced systematic reviewers (EEJ and CM) and information specialists (HOK and AS). Two editors with considerable expertise in computational and data science (CM and HOK) were responsible for the construction of the updated SR Toolbox.

However, there are some limitations of this work. The initial mapping exercise was conducted by a single reviewer, with a second checking some records for accuracy. This may be considered a bias, as it is possible that there may some minor inaccuracies in coding and charting of the tools and guidelines.

### Potential areas for future research

As part of the mapping exercise for this work, we added a column in our Excel sheet to identify when the software tool or guideline was added to the SR Toolbox. This will allow us to determine the trajectory of publications and the rate at which new software tools are being added in the future more accurately.

This column may be one way of identifying areas for expansion or refinement within future iterations of the SR Toolbox. For example, there may also be an argument to further refine the ‘Other’ category in the SR Toolbox in future updates, particularly to highlight software tools and guidance relating to network meta-analyses and prognostic reviews. A 2016 review identified 456 network meta-analyses including at least four interventions [10], suggesting that the review type is increasing in number. Prognostic reviews have been formally adopted by Cochrane, with the first two Cochrane prognostic reviews published in 2018 [11, 12], while there have also been calls for more prognostic reviews to be conducted in response to a growing amount of primary prognostic research [13].

Living systematic reviews have also been proposed as a contribution to evidence synthesis by providing high-quality reviews that are updated as new research in the area becomes available [14]. We discussed the inclusion of living systematic reviews as a standalone review category within the new iteration of the SR Toolbox, as there has been some evidence that machine learning has been used to support the production of these reviews [15], but currently the SR Toolbox does not contain any specific guidance or software tools relating to living systematic reviews. If software tools and guidelines become available for living systematic reviews, we will consider adding this review category to the Toolbox in the future.

More generally, the mapping exercise and subsequent analysis has highlighted some areas for further research and tool production. Tools and guidance to support reviews other than systematic reviews of intervention effectiveness may be needed, particularly for reviews of reviews and qualitative reviews. Additionally, there are also very few tools or guidelines relating to stakeholder engagement in the review production process. While general guidance on how to report patient and public involvement in research exists in the form of GRIPP2 [16], and the ACTIVE framework has been developed to describe stakeholder involvement in systematic reviews [17], there are currently few other frameworks or tools specifically designed to help researchers undertaking evidence syntheses to involve wider stakeholders in the process.

## Conclusion

The updated version of the SR Toolbox is designed to be an easily-navigable interface to aid researchers in finding guidance and software tools to help conduct varying forms of evidence synthesis, informed by the evolution in evidence synthesis methodologies since its inception. Our analysis of the contents of the SR Toolbox has revealed that there are specific review families and stages of the review process that are currently well-served by guidance and software but that gaps remain surrounding others. Further investigation into these gaps may help researchers to conduct other types of review in future.

## Supplementary Information

**Additional file 1: Supplementary Material.** Eligibility criteria for SR Toolbox.

## Data Availability

The datasets used and/or analysed during the current study are available from the corresponding author on reasonable request.

## Abbreviations

DTA: Diagnostic test accuracy
MEDLINE: Medical Literature Analysis and Retrieval System Online
PHP: Hypertext preprocessor
SQL: Structured Query Language
SR: Systematic review
