# Supplementary material for: The Systematic Review Toolbox: keeping up to date with tools to support evidence synthesis
Source: Syst Rev. 2022 Dec 1;11:258. doi: 10.1186/s13643-022-02122-z (PMC9713957; doi:10.1186/s13643-022-02122-z)
Supplement: Supplementary file 1 — Additional file 1: Supplementary Material. Eligibility criteria for SR Toolbox. [file 13643_2022_2122_MOESM1_ESM.docx]

**Supplementary Material – Eligibility criteria for SR Toolbox**

**Software tools**

*Included*

- Special-purpose software tools to support specific systematic review tasks or aspects of the process.
- Custom add-ons to other software (e.g. meta-analysis add-on for excel).
- Reference managers.

*Excluded*

- General-purpose systems such as word processors or spreadsheet packages.
- Commercial statistical software (e.g. Stata, SPSS etc.).
- Tools older than 10 years and not being commonly used in systematic reviews.

**Guidance documents**

*Included*

- Quality assessment / critical appraisal checklists.
- Relevant guidelines about how to perform systematic reviews.
- Reporting standards for systematic reviews.

*Excluded*

- Text books (as the focus of the SR Toolbox is on online tools easily accessible for end users).
- Tools older than 10 years and not being commonly used in systematic reviews.
